# Supplementary figures and images for: Eastern equine encephalitis virus rapidly infects and disseminates in the brain and spinal cord of cynomolgus macaques following aerosol challenge
Source: PLoS Negl Trop Dis. 2022 May 9;16(5):e0010081. doi: 10.1371/journal.pntd.0010081 (PMC9084534; doi:10.1371/journal.pntd.0010081)

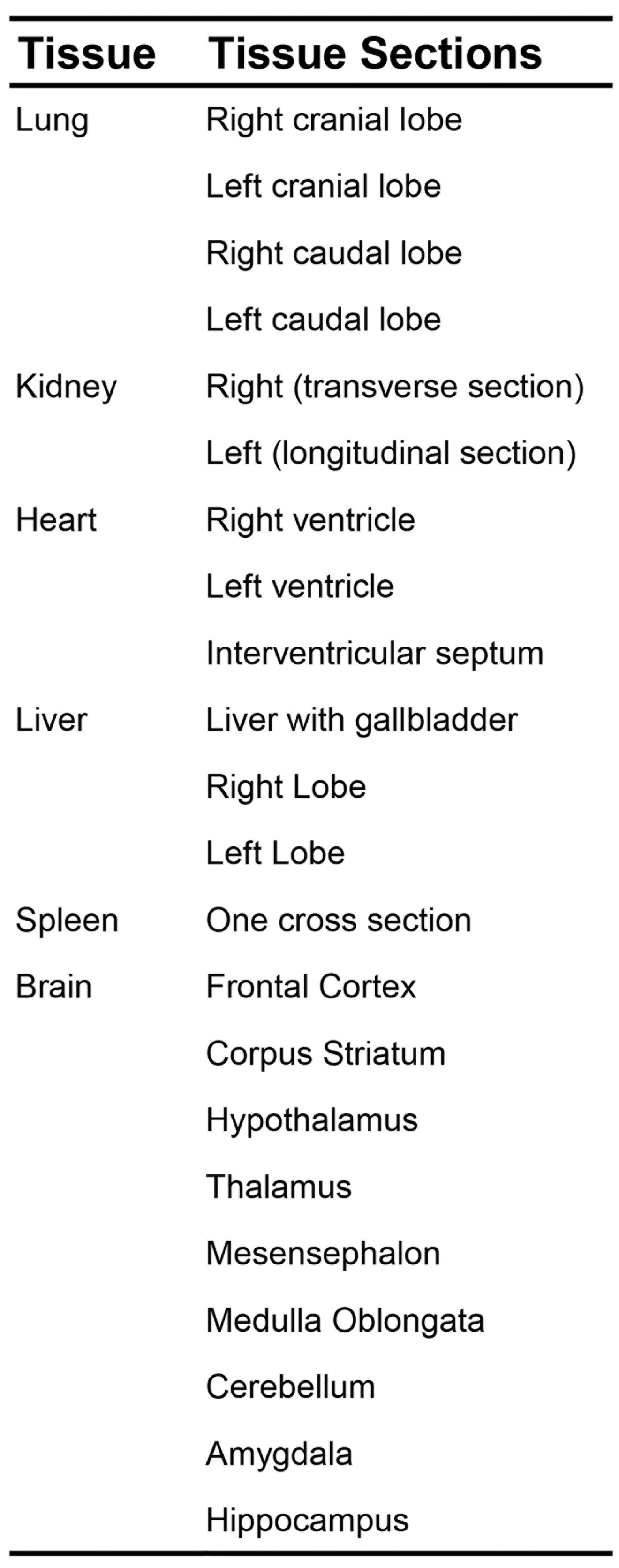

Supplement: S1 Table — (TIF) [file pntd.0010081.s008.tif]
