# Supplementary material for: Eastern equine encephalitis virus rapidly infects and disseminates in the brain and spinal cord of cynomolgus macaques following aerosol challenge
Source: PLoS Negl Trop Dis. 2022 May 9;16(5):e0010081. doi: 10.1371/journal.pntd.0010081 (PMC9084534; doi:10.1371/journal.pntd.0010081)
Supplement: Attachment — Submitted filename: Response to Reviwer Comments.docx [file pntd.0010081.s009.docx]

**Methods**


**Reviewer #1**

**Comment #1:** No the methodology is not clearly articulated. The authors mention that all details can be found in a companion manuscript that is not included here.

**Response #1:** Thank you very much for the comment. We apologize for any confusion in regard to the companion manuscript. We submitted both manuscripts as linked submissions and are not sure why the journal was not able to provide access to the companion manuscript to the reviewers. The companion manuscript has been accepted and is cited in the current manuscript. In addition, we are providing a copy of the companion with the current submission.

**Comment #2:** Line 393: the details of the virus stock should be described here as well.

**Response #2:** Thank you very much for the comment. We have added details on the virus stock (See lines 131-137).

**Comment #3:** Line 405: the study design needs to be described here as well.

**Response #3:** Thank you very much for the comment. We have added a detailed section on the NHP study design (See lines 148-162).

**Comment #4:** Line 409: what exactly does the “aerosol route” mean? Was this large or small particle aerosols? Via a nebulizer? intrabronchial instillation? Why was the specific inoculum dose chosen?

**Response #4:** Thank you very much for the comment. The aerosol route was achieved via a nebulizer. We have added a detailed section on the NHP study design (See lines 156-158).

The subcutaneous route does not produce uniform lethal disease (see reference below). In contrast, the aerosol route can produce uniform lethal disease (manuscript references 8-10). This study was designed to produce uniform severe disease in NHPs via an established aerosol route (See references 14-15).

Smith DR, Schmaljohn CS, Badger C, Ostrowski K, Zeng X, Grimes SD, Rayner JO. Comparative pathology study of Venezuelan, eastern, and western equine encephalitis viruses in non-human primates. Antiviral Res. 2020 Oct;182:104875.**:**

**Comment #5:** Histopathology figures: all histopathology figures would benefit by the inclusion of symbols or arrowheads highlighting key pathological findings. This is critically important for the nonspecialist.

**Response #5:** Thank you very much for the comment. We have added arrows in figures 2, 4, and 7.

**Reviewer #2:**

**Comment #1:** Were other methodologies considered for detecting viral replication such as staining for double-stranded RNA as a proxy for replication centers?

**Response #1:** Thank you very much for the comment. We stained for single stranded RNA to detect for the presence of EEEV genome and did not explore other methodologies such as double stranded RNA.

--------------------

**Results**

**Reviewer #1:**

**Comment #1:** Line 123: the experimental results section should include some contextualization of the experimental parameters, e.g., dose, timing, n=?, etc. That is, what exactly was done to the animals to infect them. In addition, how did you confirm productive infection? Were there daily blood draws? Does aerosol inoculation result in systemic infection? Seroconversion?

**Response #1:** Thank you very much for the comment. The questions asked by the reviewer are in the companion manuscript. We submitted both manuscripts as linked submissions and are not sure why the journal was not able to provide access to the companion manuscript to the reviewers. The companion manuscript has been accepted and is cited in the current manuscript. In addition, we are providing a copy of the companion with the current submission.

**Comment #2:** Line 125: what was the time of euthansia?

**Response #2:** Thank you very much for the comment. Please see lines 119-121 and 158-160.

**Comment #3:** Line 133: what companion manuscript?

**Response #3:** Thank you very much for the comment. The questions asked by the reviewer are in the companion manuscript. We submitted both manuscripts as linked submissions and are not sure why the journal was not able to provide access to the companion manuscript to the reviewers. The companion manuscript has been accepted and is cited in the current manuscript. In addition, we are providing a copy of the companion with the current submission.

**Comment #4:** Line 244-245: are there differences in the way virus is administered in the different animal models that could explain differences in pathology and replication?

**Response #4:** Thank you very much for the comment. We agree with the reviewer. Please see lines 391-405.

**Comment #5:** Line 226-269: it isn’t clear if any of these referenced studies were done with macaques.

**Response #5:** Thank you very much for the comment. The studies were performed in cynomolgus macaques. Please see lines 344-346.

**Comment #6:** Line 319-324: None of the telemetry data are presented in the results.

**Response #6:** Thank you very much for the comment. The questions asked by the reviewer are in the companion manuscript. We submitted both manuscripts as linked submissions and are not sure why the journal was not able to provide access to the companion manuscript to the reviewers. The companion manuscript has been accepted and is cited in the current manuscript. In addition, we are providing a copy of the companion with the current submission.

**Comment #7:** Line 381: this is the first mention of the timing of clinical signs after inoculation. This should be more thoroughly described in the results section.

**Response #7:** Thank you very much for the comment. Text has been added for clarity. Please see lines 119-121.

**Reviewer #2**

**Comment #1:** Figures (General): Since sections from control animals are unavailable for comparison, please provide callouts (arrows or otherwise) to example histology lesions described in text for additional clarity.

**Response #1:** Thank you very much for the comment. We have added arrows in figures 2, 4, and 7.

--------------------

**Conclusions**

**Comment #1:** Reviewer #1: Discussion: overall, the discussion is comprehensive but it in many places it borders on results. In addition, it was unclear in many instances whether results from the companion manuscript were simply being described again here. I suggest trying to tighten the discussion to only include the information necessary to understand the study described here.

**Response #1:** Thank you very much for the comment. The pathology studies in natural or experimental settings have been are extremely limited as demonstrated by few published studies. In the discussion we provided a comprehensive comparison between the current studies and previous finding in either natural or experiment studies.

We have clarified the text. Please see lines 373-375, 401-405, and 424-426.

**Comment #2:** Lines 278-279: Add context to expected cellular targets for infection (i.e. inhibition in myeloid cells described in Trobaugh et al. 2014 and others)

**Response #2:** Thank you very much for the comment. The manuscript is an elegant and important study on the immune evasion of EEEV in a murine model. We did not include this reference as the discussion was about EEEV localization in the CNS in previous studies.

--------------------

**Editorial and Data Presentation Modifications?**


**Reviewer #1:**

**Comment #1:** Grammar and syntax could be improved throughout and the manuscript would benefit from a careful editorial review.

**Response #1:** Thank you very much for the comment. We have addressed this throughout the manuscript.

**Reviewer #2:**

**Comment #1:** Were lymphatics (other than spleen) also examined for EEEV (RNA or antigen)?

**Response #1:** Thank you very much for the comment. Our results showed lack of pathology, viral RNA and proteins in the lung, heart, spleen, kidneys, and liver. Consequently, we did not investigate the lymphatic system.

**Comment #2:** Lines 393-394: If a citation to the companion manuscript is unavailable at the time of acceptance, please provide sufficient detail about virus stock, including passage history.

**Response #2:** Thank you very much for the comment. We have added details on the virus stock (See lines 131-137).

**Comment #3:** Lines 439-441: Please provide a citation or additional information for non-commercial reagent. What alphavirus antigen(s) was/were used to derive polyclonal antibody?

**Response #3:** Thank you very much for the comment. We have added details on the polyclonal antibody (See lines 189-192).

**Comment #4:** Lines 454-458: Please provide a citation or additional information for non-commercial reagent. Was Rabbit anti-EEEV raised against whole virus or specific antigenic regions?

**Response #4:** Thank you very much for the comment. We have added details on the polyclonal antibody (See lines 189-192).

--------------------

**Summary and General Comments**


**Reviewer #1**

**Comment #1:** Here the authors have produced a paper investigating pathogenic outcomes in crab-eating macaques that were infected with eastern equine encephalitis virus. Using aerosol inoculation, they demonstrate that the virus is found in many parts of the brain and spinal cord, and the virus gets there via the olfactory bulb. While these results are interesting and important, conclusions from this work are diminished by the lack of information presented pertaining to overall infection parameters. In addition, the authors reference a companion manuscript that contains additional details but some of these details are critical for interpretation of the work presented herein. In general, not enough information is presented on the overall study design and acute infection parameters. The work seems to be divided in the two papers strictly along the lines of pre vs. post euthanasia analyses—althought this is difficult to assess because the companion manuscript was not included. You basically need to switch back and forth between the two to manuscripts to understand what is happening, and that isn’t possible with submission of this manuscript. I am not clear on the justification for splitting the study into separate manuscripts, so unless this is clearly delineated I would strongly encourage the team to submit a single combined manuscript. At a minimum this manuscript needs to be written in a manner that it is stand alone and can be fully understood without significant references of the companion manuscript.

**Response #1:** Thank you very much for the comment. The questions asked by the reviewer are in the companion manuscript. We submitted both manuscripts as linked submissions and are not sure why the journal was not able to provide access to the companion manuscript to the reviewers. The companion manuscript has been accepted and is cited in the current manuscript. In addition, we are providing a copy of the companion with the current submission.

We have updated the manuscript to provide additional details that are critical for the interpretation of the current study. Please see above.

The two manuscripts detailing the study represent two separate scientific investigation. The first manuscript details the utilization of advance telemetry to measure critical autonomic nervous system functions. The study was first of its kind and demonstrated rapid and profound alteration of critical functions leading to the NHPs meeting the euthanasia criteria. The second (present) manuscript investigated the underlying EEEV pathogenesis mechanism/s that led to the alteration of the critical parameters in the organs and tissues of the NHPs.

**Reviewer #3**

**Comment #1:** It is difficult to follow some aspects of the results because the authors keep referring to the “companion manuscript” but no reference is provided. More details need to be provided. At a minimum the reference needs to be provided. It seems like these two manuscripts should be published together.

**Response #1:** Thank you very much for the comment. The questions asked by the reviewer are in the companion manuscript. We submitted both manuscripts as linked submissions and are not sure why the journal was not able to provide access to the companion manuscript to the reviewers. The companion manuscript has been accepted and is cited in the current manuscript. In addition, we are providing a copy of the companion with the current submission.

We have updated the manuscript to provide additional details that are critical for the interpretation of the current study. Please see above.

**Comment #2:** The number of figures needs to be reduced and should be consolidated to present/highlight major findings. For example, Figure 1 is not necessary because it shows no histopathology. For other figures, the authors should show representative images and not the image from each animal to reduce the number of figures. For example, figures 3, 4, 5, and 6 could all be combined into one figure with representative images. The same for figures 7, 8, 9. Figures 10-12 could be combined into one as well.
3.

**Response #2:** Thank you very much for the comment. We respectfully disagree with the comment for several reasons.

1) The data demonstrates that all tissues and organs of four outbred NHPs displayed uniform pathology including microscopic lesions, virus RNA and protein distribution, and detection of infectious virions in axons of neurons.
2) The study followed the extensive connections of the olfactory tract to investigate the dissemination of EEEV in the brain. These were either not investigated and/or were not reported in previous studies.
3) The visceral organs displayed similar pathology to that found in hypothalamus and spinal cord. The IHC and ISH figures for the visceral organs were removed from the main text and are supplemental figures.
4) Pathology studies in the NHPs with alphaviruses have either been limited and/or incomplete. The cost of our study with advanced telemetry in NHPs was $500k. We were extraordinarily fortunate to have the funding for perform such a cutting-edge study and perform the first comprehensive NHP pathology study following aerosol exposure. We sincerely hope that others in the future can benefit from our comprehensive pathology study.

For these reasons we sincerely hope that the reviewers will allow us to publish our figures in the current format.

**Comment #3:** Some of the major findings and statements conflict with previous reports on the pathogenesis of EEEV in NHPs and are not discussed. For example, line 244-245 states that the dissemination of EEEV following aerosol infection has not been investigated in previous macaque studies, but this is not accurate. For example, Roy et al. 2013 found high viral loads in peripheral tissues of animals that succumbed to infection on days 4-6 post-infection. Additionally, the pathology data for the Reed et al. 2007 JID article is published by Steele and Twenhafel 2010 Vet. Pathol. 47, 790—805. This article describes the key pathological features in the brain as severe meningoencephalomyelitis. These results seem to conflict with the current study where the authors’ state that “little or no pathological lesions” were observed.

**Response #3:** Thank you very much for the comment. We have added the Steele reference to the manuscript.

The current manuscript complements and extends the previous limited findings in NHPs infected via aerosol route (please see lines 344-358). However, due to the limited available data from the previous studies, only broad comparisons can be made. For example, Roy et al. states that brain and spinal cord, however, it did not state the regions examined. A paragraph comprising of five sentences and a single figure of the temporal and frontal cortex are shown. Similarly, the Steele et al. describes the pathology in of the Reed et al. in five sentences and a single table.

**Comment #4:** Also, pathogenesis studies of EEEV in NHPs by subcutaneous exposure (to mimic mosquito transmission) have been completed and should be discussed.

**Response #4:** Thank you very much for the comment. We have added the reference (Please see line 409).

**Comment #5:** The authors need to make clearer that their findings are specifically for animals who have been exposed to EEEV by aerosol. For example, this could be clarified in lines 227-228 and 242-243.

**Response #5:** Thank you very much for the comment. Clarification has been added to text. Please see lines 359-361.

**Comment #6:** It would be beneficial if the authors would expand their discussion about what is known about the pathogenesis of EEEV primarily in humans (not lump all mammalian species as a whole; lines 257-258) and how their results compare. For example, vasculitis is an important pathological feature in humans infected with EEEV and have been reported in other aerosol exposure studies of EEEV in cynomolgus macaques, but I do not see it discussed in the current study. Why does it seem that the current study has different conclusions/findings compared to other aerosol exposure studies in cynomolgus macaques that did observe significant pathology in the brain?

**Response #6:** Thank you very much for the comment. The human data is extremely limited and the route of infection is via a mosquito bite. In addition, the studies in NHPs and small animal models have shown that the route of infection can significantly alter virus pathogenesis. Our study is in agreement with the latter. Consequently, a broader discussion with the aerosol route vs. natural infection was discussed.

**Comment #7:** Comment #Line 135: Shouldn’t this be reported as PFU/g since it is the titer in tissue?

**Response #7:** Thank you very much for the comment. The error has been corrected (see line 253).

**Comment #8:** Line 139: Should “vacuolation of the neutrophil” be vacuolation of the neuropil?

**Response #8:** Thank you very much for the comment. The error has been corrected (see line 258).

**Comment #9:** All H&E images: Arrows should be added to highlight findings (i.e. necrosis, neutrophilic infiltrates, etc) for readers that are not used to looking at histopathology images.

**Response #9:** Thank you very much for the comment. We have added arrows in figures 2, 4, and 7.
