# Supplementary material for: Eastern equine encephalitis virus rapidly infects and disseminates in the brain and spinal cord of cynomolgus macaques following aerosol challenge
Source: PLoS Negl Trop Dis. 2022 May 9;16(5):e0010081. doi: 10.1371/journal.pntd.0010081 (PMC9084534; doi:10.1371/journal.pntd.0010081)
Supplement: Attachment — Submitted filename: Responses to Reviewer Comments.docx [file pntd.0010081.s010.docx]

Reviewer's Responses to Questions

**Methods**

***Reviewer #4***

**Comment #1:** Reviewer #4: The objectives of the study are articulated but they should explain why the effort is limited to aerosol exposure only.

**Response #1:** Thank you very much for the comment. The subcutaneous route does not produce uniform lethal disease (see reference below). In contrast, the aerosol route can produce uniform lethal disease (manuscript references 10-13). This study was designed to produce uniform severe disease in NHPs via an established aerosol route (See references 10-13).

Smith DR, Schmaljohn CS, Badger C, Ostrowski K, Zeng X, Grimes SD, Rayner JO. Comparative pathology study of Venezuelan, eastern, and western equine encephalitis viruses in non-human primates. Antiviral Res. 2020 Oct;182:104875.

**Comment #2:** This reviewer questions if a detailed analysis of pathology from aerosol exposure with EEE gives a thorough picture of EEE pathology in NHPs.

**Response #2:** Thank you very much for the comment. The aerosol route enables direct access to the brain and provides insights into how rapidly EEEV can spread in the brain and spinal cord to cause severe clinical disease. However, the underlying mechanism of severe disease is not due to substantial pathology and/or host immune response/s but is rather due to neuronal dysfunction. Pathology data for EEEV infection via mosquito bite is extremely limited. The kinetics of the infection and subsequent clinical disease via mosquito route will be slower and the host immune response and viral and/or host induced pathology will play a considerable role in pathogenesis. Future studies should investigate such hypotheses.

**Comment #3:** What is the power calculation for the sample size [number of macaques (4)] used in the study to analyze data significance? How was this number determined? I realize NHP studies are costly.

**Response #3:** Thank you very much for the comment. The aerosol route can produce uniform lethal disease (manuscript references 10-13). Consequently, the study designed with 80% power to detect alteration in the physiological parameters. There were a number of factors that were considered for the study and are outlined below:

1) Comprehensive advanced telemetry had not been used in high containment previously. Our group overcame enormous technical challenges and implanted animals with devices to enabled continuous and simultaneous measurement of multiple clinical parameters relevant to human disease.
2) This was an extraordinary expensive study. We were very fortunate to have obtained funding from the Department of Defense to carry out such state-of-the-art study. The cost of purchasing each NHP was $6,000. In addition, the telemetry devices and the cost of implantation was an additional $24,000 per NHP.
3) Previous studies had measured four parameters (clinical disease, viremia, temperature, and survival) per NHP to study EEEV disease. Our study aimed to provide seven additional parameters (respiration, activity, alteration in NHP behavior, heart rate, blood pressure, ECG, and EEG) per NHP. This technology enables a comprehensive evaluation of critical physiological parameters.
4) The study was designed as a pilot study of 4 NHPs to investigate alteration of critical physiological parameters utilizing advance telemetry.

***Reviewer #5***

**Comment #1:** Methods of the manuscript are acceptable; however, alternative approaches that might improve the capacity for detection of replicating virus such as in situ hybridization for negative strand RNA would have been useful rather than reporting cytopathic vacuoles. Branched chain amplification coupled with RNAscope should be extremely sensitive for detection of replication intermediates.

**Response #1:** Thank you very much for the comment. Negative strand in produced 1,000 to 10,000 times lower than the positive strand. We focused our study to detect positive strand to ensure the detection throughout the brain, spinal cord, and visceral organs. We are investigating the tissues further and will explore the suggested method.

**Comment #2:** Directly relevant to the hypothesized mechanism of disease progression (neuronal dysfunction) have the investigators examined means for characterizing this with specifically? Quantifying possible axonomal degeneration or some other marker for neuronal function?

**Response #2:** Thank you very much for the comment. This is the first study to show neuronal dysfunction as a potential mechanism that underlies EEEV pathogenesis. We are in the process of performing additional studies to further investigate this mechanism and will be publishing additional manuscripts.

--------------------

**Results**

***Reviewer #4***

**Comment #1:** Otherwise, this work provides pathogenesis details not addressed in previous EEEV challenges following aerosol exposures. Having 21 figures and 5 supplemental figures makes for a rather (figure) dense paper and this reviewer considers it important where possible to consolidate data presentation critical for this paper or at least move some of the paper figures to the supplemental section.

**Response #1:** Thank you very much for the comment. Figures 20 and 21 have been removed from the main manuscript and are now part of the supplemental figures.

***Reviewer #5***

**Comment #1:** Results presented are adequate but I would have liked to have seen some specific data regarding infiltrates and a rigorous assessment of whether EEEV RNA was identified. This addresses the central hypothesis generated and could also be related to reduced inflammation due to a lack of peripheral replication due to the route of exposure.

**Response #1:** Thank you very much for the comment. The infiltrates were observed, however, they were limited to amygdala and hippocampus (see lines 248-251). The infiltrates were either minimal or lacking in many parts of the CNS that were investigated.

**Conclusions**

***Reviewer #4***

**Comment #1:** Others have already shown encephalitic alphaviruses can gain entry to the CNS via the olfactory tract following intranasal or aerosol routes of challenge with neurovirulent alphaviruses in animal models. However, the studies by Williams et al. show little (to no) such evidence of virus replication outside the CNS as well as in the brain. This is a curious finding. Encephalitic alphaviruses (WEEV and VEEV) definitely show virus replication in the brain of mice following intranasal and peripheral routes of challenge but the authors here show minimal evidence of viral replication in macaque CNS. This is addressed in the Discussion but only in general terms and may highlight viral pathogenesis differences using different animal models.

**Response #1:** Thank you very much for the comment. We agree with the reviewer as considerable work remains to be performed to better understand the differences in the animal models. Relevant discussion was limited to mice, guinea pigs, and marmosets following aerosol challenge (See lines 365-376).

***Reviewer #5***

**Comment #1:** Necrotic lesions, although reported to be very rare in this study, could significantly contribute to disease progression. The authors should indicate this as a possibility within the Discussion section.

**Response #1:** Thank you very much for the comment. The comment has been incorporated (see lines 462-464).

**Comment #2:** More specific discussion regarding this route of infection should be made for interpretation of the results presented herein with previous reports. The lack of peripheral replication could have a significant impact on inducing innate immune responses and subsequent inflammation within the CNS.

**Response #2:** Thank you very much for the comment. Pathology data in animal models following aerosol challenge is extremely limited. We have discussed the relevant data in lines 365-375.

--------------------

Editorial and Data Presentation Modifications

**Reviewer #5**

**Comment #1:** The authors refer to a North American lineage virus. Functionally, EEEV is the only virus within this group after the South American lineage viruses were reclassified as Madriagas virus.

**Response #1:** Thank you very much for the comment. The comment has been incorporated (see line 332).

Summary and General Comments

***Reviewer #4***

**Comment #1:** PTND-20-02124R1: This work by Williams et.al. sheds light on EEEV pathogenesis in the CNS of an NHP (macaques) following aerosol exposure of EEEV. The team used advanced telemetry to measure the animal’s physiological parameters following aerosol exposure. Others have already shown encephalitic alphaviruses can gain entry to the CNS via the olfactory tract following intranasal or aerosol routes of challenge with neurovirulent alphaviruses in animal models. However, the studies by Williams et al. show little (to no) such evidence of virus replication outside the CNS as well as in the brain. Encephalitic alphaviruses (WEEV and VEEV) definitely show virus replication in the brain of mice following intranasal and peripheral routes of challenge but the authors here show minimal evidence in macaques. This is addressed in the Discussion but only in general terms but may highlight pathogenesis differences using different animal models. Additionally, outside of possible

exposures through bioterrorist events or laboratory accidents, does it make sense to report aerosol exposures only and pass on any attempts to define or describe outcomes of virus pathology/challenge from peripheral exposures (the current danger from mosquito-borne alphaviruses). I realize NHP studies are expensive but some of the claims reported here appear to contradict previous pathogenesis studies in NHP and other animal models and there is minimal discussion of this point. Are the authors assuming the pathologies will be the same for aerosol vs peripheral exposures in NHPs?

**Response#1:** Thank you very much for the comment. The previous NHP studies performed limited pathology and therefore a comprehensive comparison with the present study cannot be performed. Roy et al. states that brain and spinal cord, however, it did not state the regions examined. A paragraph comprising of five sentences and a single figure of the temporal and frontal cortex are shown. Similarly, the Steele et al. describes the pathology in of the Reed et al. in five sentences and a single table.

We do not believe that pathology following aerosol will be different than the natural route and have addressed this in the discussion. We performed a comprehensive literature search of natural infections in many different species including humans and summarized/discussed the findings in lines 377-390.
